# Sexual hallucinations and delusions in borderline personality disorder

**DOI:** 10.3389/fpsyt.2026.1750041

**Published:** 2026-02-25

**Authors:** Rosemarij J.B. van Veen, Jan Dirk Blom, Emma H.C. van Rooijen, Ingmar H.A. Franken, Christina W. Slotema

**Affiliations:** 1 Parnassia Academy, Parnassia Psychiatric Institute, The Hague, Netherlands; 2 Department of Psychology, Education, and Child Studies, Erasmus University Rotterdam, Rotterdam, Netherlands; 3 Institute of Psychology, Leiden University, Leiden, Netherlands; 4 Department of Psychiatry, University of Groningen, Groningen, Netherlands; 5 Department of Pediatric Medicine, Leiden University Medical Centre, Leiden, Netherlands

**Keywords:** borderline personality disorder, childhood trauma, multimodal hallucination, prevalence, sexual abuse, sexual delusion, sexual hallucination

## Abstract

**Introduction:**

Sexual hallucinations have lifetime prevalence rates of 0.4% in the general population and up to 44% in schizophrenia spectrum disorders. For borderline personality disorder (BPD) these rates are unknown. We therefore studied prevalence rates of sexual hallucinations and delusions in this group, as well as their phenomenological characteristics and their connection with childhood trauma.

**Methods:**

We carried out a cross-sectional study among outpatients with BPD, using the Sexual Hallucinations and Delusions Questionnaire (SHDQ), the Psychotic Symptom Rating Scales–Auditory Verbal Hallucinations (PSYRATS-AVH) and the Childhood Trauma Questionnaire (CTQ).

**Results:**

Among 81 people interviewed, we found a lifetime prevalence rate of 59.3% for sexual hallucinations, and 49.4% for sexual delusions. For the last month these rates were 36.0% and 31.0%, respectively. In this group somatic, visual, tactile and auditory sexual hallucinations were more common than olfactory and gustatory ones. Childhood trauma was reported by all participants, with emotional abuse and sexual abuse showing the strongest link with sexual hallucinations and delusions.

**Discussion:**

Sexual hallucinations and delusions are very common in people with BPD. They may well explain (part of) the emotional and behavioral aspects considered characteristic of BPD. Our finding that childhood trauma was reported by all participants indicates that more research is needed to explore the impact of trauma in this specific population. We also provide recommendations for clinical practice, including treatment options.

## Introduction

Sexual hallucinations are intrusive phenomena that can be experienced in various sensory modalities (1, 2). For example, people may experience orgastic sensations, touch of erogenous zones, or images with an explicit sexual content in the absence of corresponding stimuli from the outside world. Although valued positively by a small minority of people (notably those experiencing auras in the context of epilepsy) they are mostly unwanted and burdening (2). They are not as rare as previously thought, with estimated lifetime prevalence rates of 0.4% in the general population and up to 44% in chronically hospitalized patients with schizophrenia spectrum disorders (2, 3). In addition to their association with the latter group of disorders, they are chiefly reported on in the context of sleep paralysis, epilepsy, narcolepsy, sedative use and pelvic pathology (e.g. Tarlov cysts, clitoral engorgement (2), whether or not in combination with sexual delusions. Studies have established an association between sexual hallucinations, sexual delusions and childhood trauma with odds ratios of up to 8.7 in patients with schizophrenia spectrum disorders (1, 3). As far as we know, there are no published reports on these phenomena in people diagnosed with borderline personality disorder (BPD), a condition that is also strongly associated with childhood trauma. One reason for this omission may be that hallucinations in BPD have long been considered infrequent, mild and transient in nature (4–6). However, several studies report a lifetime prevalence of hallucinations in general in up to 60% of this group (4, 5) and moreover indicate that hallucinations in this group are often indistinguishable from those in schizophrenia spectrum disorders. For example, verbal auditory hallucinations in both groups share the same phenomenological characteristics (7, 8). In both groups, these hallucinations have also tended to be experienced for years, albeit with longer-lasting symptom-free intervals in BPD, and yet with ensuing levels of distress that are often markedly higher than in people with schizophrenia spectrum disorder (5, 7, 9, 10). Despite these empirical findings, major classifications such as the *Diagnostic and Statistical Manual of Mental Disorders* (DSM-5) and the *International Classification of Diseases* (ICD-11) do not mention hallucinations as diagnostic criteria for BPD (11, 12). This is probably a second reason why hallucinations in general, but sexual hallucinations too, have long been overlooked in this group. This could be another reason why not only people experiencing these phenomena may be reluctant to discuss them, but also the health professionals to whom they turn for help.

In line with previous findings for people with schizophrenia spectrum disorders, hallucinations in people with BPD are often associated with childhood trauma (5). Prevalence rates reported for childhood trauma in BPD are up to 93%, with rates for sexual abuse varying from 40% to 71% (13–15). Given these figures, we also expect high rates of sexual hallucinations and delusions in the group diagnosed with BPD. Therefore, this study aims to answer the following research questions:

1. What are the present-state and lifetime prevalence rates of sexual hallucinations and delusions in people diagnosed with BPD?
2. What are the phenomenological characteristics of these symptoms?
3. What are the prevalence rates, frequency, and burden of hallucinations in general in this group?
4. Is there a relationship between childhood trauma and sexual hallucinations and delusions?

## Materials and methods

### Study design

We conducted a cross-sectional study among people at an outpatient clinic for personality disorders at Parnassia Psychiatric Institute, The Hague. The study received approval from the Ethical Review Board of Leiden University Medical Center (number: NL66211.058.18) and was conducted from 2019 through 2023. All participants provided written informed consent.

### Participants

People were included if they fulfilled the DSM-5 diagnostic criteria for BPD, were ≥ 18 years of age, were able to provide informed consent, and had a sufficient command of the Dutch or English language. They were excluded if they had a comorbid diagnosis of schizophrenia spectrum disorder (save the unspecified variant) or if their therapist considered them unsuitable for participation, e.g. due to a current crisis. Other comorbid diagnoses were no exclusion criterion, nor was medication use. Recruitment took place via health professionals, posters in waiting rooms, and phone calls to people on a waiting list for the outpatient clinic for personality disorders who had given permission to be approached for research purposes. When interested, people were informed by their treating health professional that the study entailed being interviewed with the aid of questionnaires for people diagnosed with BPD, on hallucinations and other unusual experiences, including sexually themed ones, and on childhood trauma. The health professionals involved had been instructed to emphasize that the aim of the study was to gain insight into prevalence rates and that participation was therefore valuable, notwithstanding whether one would recognize such experiences oneself.

### Procedures

We collected demographic data and phenomenological as well as information on medication use and duration of treatment. Comorbid diagnoses were retrieved from the participants’ medical records. The presence and nature of sexual hallucinations and delusions were explored by making use of the *Sexual Hallucinations and Delusions Questionnaire* (SHDQ), a semi-structured, non-validated questionnaire developed by our group, which comprises 27 questions on the presence of sexual hallucinations and delusions (1) (**Supplementary Material 1**). We defined sexual hallucinations as ‘sexually explicit perceptions experienced during wakefulness in the absence of exogenous or physiological endogenous triggers’, and sexual delusions as ‘false beliefs of a sexual nature, held despite incontrovertible evidence to the contrary’. When dealing with dissociative flashbacks, we made sure to distinguish between recollections and reperceptions of prior traumatic events, and accept only the latter as hallucinations. All phenomena were scored as being either ‘absent’, ‘present during the last month’ (i.e. ‘present state’), or ‘present in lifetime’. Written reports on these data were subsequently discussed with an experienced clinical psychiatrist (the second author). To explore the prevalence, frequency and burden of auditory, visual, tactile, olfactory, and gustatory hallucinations, we used the following subscales of the *Psychotic Symptom Rating Scales–Auditory Verbal Hallucinations* [PSYRATS-AVH (16)]: *frequency*, *amount of negative content*, *amount of distress*, and *disruption to life*. To assess the presence of childhood trauma, we used the Dutch version of the *Childhood Trauma Questionnaire* (CTQ (17)), which contains 27 questions about physical abuse and neglect, emotional abuse and neglect, and sexual abuse, all before the age of 18. Results were scored on a five-point Likert scale (ranging from ‘never true’ to ‘very often true’). All interviews were performed by trained psychologists, psychiatrists, and psychiatric residents.

### Statistical analyses

All statistical analyses were conducted with the aid of IBM SPSS Statistics version 27. Present-state and lifetime prevalence rates of (sexual) hallucinations, delusions, and childhood trauma are reported in absolute numbers and percentages. Other outcomes of the questionnaires are reported in medians/means and their standard deviations (SD). A logistic regression analysis was conducted to explore the effects of the specific types of childhood trauma on sexual hallucinations and delusions.

## Results

We included 81 people with a DSM diagnosis of BPD, 88% of whom identified as female, 11% as male, and 1% as ‘other’. The majority of the participants had finished secondary school or a higher type of education, and half of them were currently employed. Depressive disorder and PTSD were the most commonly diagnosed comorbid disorders. Of all participants 84% used psychotropic medication, the largest group of which (used by almost 50%) was antidepressants. This, along with further demographic information, is reflected in **Table 1**.

**Table 1:** Demographic data (N = 81).

**Age, mean (SD)**		32.15 years (11.38 years)
**Gender, n (%)**	Female	71 (87.7%)
	Male	9 (11.1%)
	Other	1 (1.2%)
**Ethnicity n (%)**	Dutch (both parents Dutch)	37 (45.7%)
	Dutch (one parent non-Dutch)	28 (34.6%)
	Non-Dutch	16 (19.8%)
**Education, n (%)**	Elementary or secondary school	12 (14.8%)
	Associate’s degree	35 (43.2%)
	College degree	21 (25.9%)
	University degree	13 (16.0%)
**In a relationship, n (%)**		48 (59.3%)
**Employed, n (%)**		41 (50.6%)
**Months in treatment, mean (SD) Psychotropic medication, total**		50.5 months (29.77 months)
**On medication, n (%)**		68 (84.0%)
	Antidepressant	40 (49.4%)
	Antipsychotic	15 (18.5%)
	Sedative	29 (35.8%)
	Stimulant	12 (14.8%)
	Other	39 (48.5%)
**Comorbidities, n (%)**	Anxiety disorder	14 (17.3%)
	Depressive disorder	31 (38.3%)
	PTSD	19 (23.5%)
	Other personality disorder	16 (19.8%)
	Eating disorder	3 (3.7%)
	ADHD	13 (16.0%)
	Substance use disorder (excluding tobacco)	3 (3.7%)
	Unspecified schizophrenia spectrum disorder or other psychotic disorder (formerly known as psychotic disorder NOS)	1 (1.2%)
	Bipolar disorder	1 (1.2%)

Within this group, the lifetime prevalence rate for sexual hallucinations was 59.3%; for the present state, this was 35.8% (**Table 2**). For sexual delusions, the lifetime and present-state prevalence rates were respectively 49.4% and 30.9%. Most prevalent were delusions of reference. All participants reported on having experienced some type of childhood trauma. **Table 3** provides an overview of total and mean scores on the CTQ per type of childhood trauma, as well as scores for the groups that had reported on hallucinations in general, sexual hallucinations, sexual delusions, and having had no (prior experience with) hallucinations.

**Table 2:** Prevalence rates of sexual hallucinations and delusions.

	Present state, n (%)	Lifetime, n (%)
Type of sexual hallucination		
Any	29 (35.8%)	48 (59.3%)
Auditory (including verbal auditory)	9 (11.1%)	17 (21.0%)
Visual	12 (16.0%)	27 (33.3%)
Tactile	10 (12.3%)	17 (21.0%)
Somatic	22 (27.2%)	30 (37.0%)
Olfactory	4 (4.9%)	10 (12.3%)
Gustatory	1 (1.2%)	4 (5.0%)
Type of sexual delusion		
Any	25 (30.9%)	40 (49.4%)
Reference	21 (25.9%)	29 (35.8%)
Somatic	6 (7.4%)	13 (16.0%)
Jealousy	6 (7.4%)	17 (11.0%)

**Table 3:** CTQ scores for people reporting hallucinations in general, sexual hallucinations, and sexual delusions.

	N	Total, mean (SD)	Physical neglect, mean (SD)	Physical abuse, mean (SD)	Emotional neglect, mean (SD)	Emotional abuse, mean (SD)	Sexual abuse, mean (SD)
Total sample	81	67.3 (19.6)	10.2 (4.8)	10.9 (5.9)	17.4 (5.5)	17.2 (5.3)	10.7 (5.6)
Hallucinations absent	13	69.3 (17.9)	11.8 (6.3)	10.2 (6.8)	20.3 (3.7)	17.8 (4.0)	9.0 (5.2)
Hallucinations present	46	69.3 (18.3)	10.7 (4.2)	11.1 (5.7)	16.9 (5.0)	17.3 (5.2)	12.0 (5.3)
Sexual hallucinations absent	33	62.9 (18.2)	9.8 (5.1)	10.1 (5.6)	17.2 (5.6)	15.5 (4.8)	9.3 (4.9)
Sexual hallucinations present	29	72.3 (20.3)	10.8 (4.4)	11.8 (6.3)	17.5 (5.3)	19.0 (5.3)	12.2 (6.0)
Sexual delusions absent	41	60.5 (18.2)	9.6 (4.4)	9.9 (5.1)	16.3 (5.8)	15.4 (5.3)	8.3 (4.9)
Sexual delusions present	25	77.8 (13.8)	11.6 (4.3)	12.2 (6.4)	19.4 (2.6)	20.1 (3.8)	13.1 (4.9)

CTQ, Childhood Trauma Questionnaire.

Common examples of auditory sexual hallucinations were the voice of an abuser, sexually themed commands or comments, and hearing footsteps. Visual sexual hallucinations typically involved seeing the face or shape of an abuser, seeing people having sexual intercourse or seeing a vibrator. As for tactile sexual hallucinations, participants often reported feeling touch to erogenous zones or having the sensation of being sexually abused. Somatic sexual hallucinations included unwanted sexual arousal, random orgasmic sensations, sexual arousal in unexpected body parts (e.g. tips of fingers, or the back), perceived abnormal changes to the size or shape of genital organs and the sensation of being penetrated. Olfactory sexual hallucinations often involved the smell of genital odors or condoms, while gustatory ones typically involved the taste of sperm.

Sexual delusions frequently involved firmly believing in being watched in the shower or while undressing, third parties having sexual intentions, being followed for sexual reasons, feelings of jealousy or distrust about the faithfulness of one’s partner, disproportionate changes to the size or shape of genitalia and transgenderism.

As for all types of hallucination in the six main sensory modalities (whether of a sexual nature or not), the lifetime prevalence rate in the study group was 84%, and for the present state 57% (**Table 4**). Somatic, visual, tactile and auditory sexual hallucinations were more common than olfactory and gustatory ones. Most frequent were auditory hallucinations, experienced at a mean rate of once a week to daily. The highest reported levels of distress were associated with verbal auditory and tactile hallucinations with a median score of 3 indicating ‘considerable distress’ (with ensuing feelings of anxiety, nervousness, and depression), followed by visual hallucinations (some to considerable distress). Among these phenomena, verbal auditory hallucinations had the highest impact on people’s daily functioning, with an average score between 1 ‘minimal disruption to daily life’ (disturbances to some specific activities) and 2 ‘moderate disruptions to life’ (disturbances to multiple activities). People rated verbal auditory, visual and tactile hallucinations more often as negative, whereas they rated nonverbal auditory, gustatory and olfactory hallucinations more often as neutral or positive.

**Table 4:** PSYRATS scores for hallucinations per sensory modality.

	Auditory	Verbal auditory	Visual	Tactile	Gustatory	Olfactory	Any
Lifetime, n (%)	41 (50.6)	40 (49.4)	51 (63.0)	30 (37.0)	12 (15.0)	22 (27.2)	68 (84.0)
Present, n (%)	24 (29.6)	21 (25.9)	27 (33.3)	15 (18.5)	10 (12.5)	12 (14.8)	46 (56.8)
Frequency, mean (SD)median	1.79 (1.06) 1	2.24 (1.41) 2	2.00 (1.21) 2	1.87 (0.74) 2	2.10 (0.74) 2	1.75 (0.87) 2	1.30 (0.97) 1
Negativity of content, mean (SD)median	2.04 (2.26) 1	2.76 (2.26) 4	2.46 (2.10) 2.5	3.33 (2.23) 5	3.30 (2.36) 5	2.33 (1.97) 2.5	2.42 (1.70) 2.5
Ensuing distress, mean (SD)median	2.09 (1.31) 2	2.45 (1.23) 3	2.27 (1.46) 2	2.27 (1.62) 3	0.80 (0.79) 1	1.58 (1.31) 1.5	1.60 (1.26) 1.67
Impact on daily functioning,mean (SD)median	0.96 (1.16) 1	1.48 (1.57) 1	0.96 (1.25) 0	0.93 (1.22) 1	0.10 (0.32) 0	0.25 (0.62) 0	0.638 (0.80) 0.29

PSYRATS, Psychotic Symptom Rating Scales.

To explore the effects for the five subscales of childhood trauma on sexual hallucinations and delusions we conducted a logistic regression analysis. The results of the model with the childhood trauma subscales were statistically significant for sexual hallucinations and delusions (*X^2^*(5) = 16.850, *p* = 0.005 and *X^2^*(5) = 25.114, *p* = 0.000) and explained respectively 31.8% and 43.1% (Nagelkerke R^2^) of the variance (see **Table 5** for the odds ratios). Participants who had high scores on the subscales of childhood emotional abuse and sexual abuse were more likely to experience sexual hallucinations and delusions. In the case of sexual delusions, this result also held for the total CTQ score. Interestingly, we found a significant result in the opposite direction for emotional neglect and sexual hallucinations. Upon further examination, there seemed to be a spurious effect for emotional abuse, in the sense that emotional neglect only showed an effect when emotional abuse was also included in the analysis. In analyses that did not consider emotional neglect, the effect of emotional abuse still stands. This phenomenon may be attributable to the correlation between emotional neglect and emotional abuse (r = 0.640). Upon visual examination of the mean scores of emotional abuse and emotional neglect, emotional abuse had a higher score in the group of participants who had sexual hallucinations than in the group who had not, with emotional neglect showing similar scores for both groups. Likewise, upon examination of correlations, we found a positive correlation for emotional abuse and sexual hallucinations (r = 0.336), whereas this was not the case for emotional neglect and sexual hallucinations (r = 0.022).

**Table 5:** Odds ratios for sexual hallucinations in relation to various types of childhood trauma.

	Sexual hallucinations		Sexual delusions	
	Odds ratio (95% CI)	p-value	Odds ratio (95% CI)	p-value
Physical neglect	1.080 (0.910-1.1282)	0.379	1.020 (0.855-1.218)	0.826
Physical abuse	0.986 (0.864-1.125)	0.832	0.941 (0.821-1.078)	0.381
Emotional neglect	0.803 (0.669-0.964)	0.019	1.023 (0.827-1.265)	0.836
Emotional abuse	1.310 (1.092-1.571)	0.004	1.277 (1.054-1.547)	0.013
Sexual abuse	1.121(1.001-1.256)	0.049	1.197 (1.064-1.346)	0.003
CTQ total score	1.027 (0.999-1.055)	0.063	1.066 (1.026-1.106)	0.001

## Discussion

Our study of 81 outpatients diagnosed with BPD yields prevalence rates for hallucinations in general that are comparable to those reported on in previous studies (4, 5, 8, 18, 19), with the exception of visual, olfactory, and tactile hallucinations, which were slightly more prevalent in our sample than in other studies (20–22). The reported levels of distress due to hallucinations were comparable to the relatively high rates reported earlier, notably for auditory hallucinations in BPD (7, 8, 18, 24–27). More importantly though, more than a third of the participants in our study experienced sexual hallucinations during the last month, and almost 60% at some point throughout their lives, with somatic sexual hallucinations being the most prevalent type. Sexual delusions were reported on by almost a third of the participants in the last month, compared to roughly half the group during their lifetime. These rates are substantially higher than those reported on in the context of psychosis, with the lifetime prevalence rate for sexual hallucinations in our group being 1.4 times higher than in chronically hospitalized people with schizophrenia spectrum disorder (3), and 150 times higher than the rates known for the general population. For sexual delusions, such a comparison was harder to make due to a lack of studies in this field. However, compared to the rate of 15.2% for ‘attenuated or subthreshold psychotic symptoms with sexual content’ in an outpatient group with an ultra-high risk of psychosis (23), sexual delusions in our BPD group were more than twice as prevalent.

Although all participants reported childhood trauma, particularly childhood emotional abuse and sexual abuse increased the chance of experiencing sexual hallucinations and delusions. These results are in line with the literature on BPD (13–15) and with similar studies on sexual hallucinations and delusions in schizophrenia spectrum disorders (1, 3, 28). The smaller odds ratios that we report here might be explained by the high prevalence of childhood trauma in this study’s BPD population.

### Implications for the conceptualisation of BPD

Our findings challenge the traditional view that hallucinations in BPD are rare, brief, and self-limiting (11). On the contrary, they indicate that hallucinations in multiple sensory modalities are frequent in this group, and that sexual hallucinations make up a substantial proportion of them. Likewise, sexual delusions are far from rare in BPD. Our findings also challenge the defining characteristics of BPD, a condition that is traditionally operationalized along the lines of ‘a pervasive pattern of instability of interpersonal relationships, self-image, and affects, and marked impulsivity beginning by early adulthood’ (11), without any mention of hallucinations. The prevalence rates presented here are in need of replication, but if they turn out to be generalizable, they have implications for the way we operationalize BPD and diagnose and treat people with this disorder. For example, it is not unthinkable that sexual hallucinations and delusions may aggravate the emotions and behavior considered characteristic of BPD, in the sense that people who experience these phenomena may be so overwhelmed by them that they cannot help but act on them. Our findings also raise the important question of whether sexual hallucinations and delusions in this group can be prevented by preventing childhood trauma. Even though our study does not allow for causal inferences, the finding that childhood trauma was reported on by all participants is suggestive of an important etiological or at least pathoplastic role. Of note, our finding that especially emotional abuse and sexual abuse in childhood are predictors for sexual hallucinations and delusions does not imply that other types of childhood trauma are less harmful.

### Implications for clinical practice

What is needed first and foremost in clinical practice, is to alert health professionals, policy makers, and the general population of the high prevalence rate of sexual hallucinations and delusions in BPD. These phenomena should be opened up for discussion - and their high prevalence rate acknowledged - to reduce stigmatization and patient delay and to improve diagnostic procedures. Since sexual hallucinations have also been described in the context of other disorders, psychiatric as well as somatic, other potential causes should always be carefully ruled out (2). This may entail auxiliary investigations such as blood work, neuroimaging, an electroencephalogram, and - only when indicated, and with due caution - a gynaecological examination. Evidence-based treatment guidelines for sexual hallucinations have not yet been developed, and neither for the treatment of hallucinations in general in BPD (2, 29). However, based on the similarities of these symptoms with those in schizophrenia spectrum disorders, one may consider treating them in accordance with existing treatment guidelines for psychosis, especially when levels of ensuing distress are high. Randomized controlled trials, open-label pharmacological studies, and small case series suggest that even relatively low dosages of antipsychotics may be effective in this group (9, 30). Finally, cognitive-behavioral therapy and trauma-focused therapy may be effective (9, 30).

### Implications for research

Replication of our findings is a priority. Our results also warrant further research into i) other risk factors for sexual hallucinations and delusions, ii) the possible role of sensory deprivation (i.e. lack of physiological sexual stimulation) in the mediation of sexual hallucinations, iii) levels of ensuing distress, iv) neurobiological correlates and v) pharmacological and nonpharmacological treatment methods. This holds true for populations diagnosed with BPD, but by extension, also for other groups where these phenomena are prevalent (2). Last but not least, more research is needed into ways to prevent and address childhood trauma.

### Limitations

The scope of our study is confined in various ways. Firstly, due to our recruitment method there may have been selection bias, leading to an overrepresentation of people with (sexual) hallucinations and delusions. Since the patients’ health professionals were the first point of contact for anyone willing to be recruited, we moreover have no exact data on screening and exclusion figures. Nonetheless, the prevalence rates that we found for hallucinations in general were comparable to those in earlier studies on BPD. This indicates that people experiencing these phenomena were not overrepresented. Secondly, since our sample size was relatively small, replication of the present study is needed in larger groups and preferably in different institutions. Thirdly, the lack of a control group limits the generalizability of our results. Fourthly, we had relatively few male participants. BPD is more prevalent in females, but comparing the experiences of females with those of males is a topic for future research. Fifthly, the questionnaire that we deployed to assess the presence and characteristics of hallucinations and delusions (SHDQ) had not been validated. Sixthly, we were unable to establish the burden caused specifically by sexual hallucinations since the PSYRATS does not differentiate between sexual hallucinations and those of a nonsexual nature. Seventhly, we did not assess the influence of potential confounding factors such as comorbid disorders and the use of illicit substances or medications. Of note, medication use might be correlated at once with higher prevalence rates for the present state (due to increased insight) and lower rates (due to a decrease in symptomatology). And lastly, as with delusions in general, it was not always certain whether answers to our queries should count as delusions (e.g. in having the feeling of being sexualized or being cheated on).

## Conclusions

In our cross-sectional study of 81 outpatients diagnosed with borderline personality disorder, 60% reported having experienced sexual hallucinations, and 36% during the past month. For sexual delusions these rates were 49% and 31%, respectively. These rates are substantially higher than in other patient groups, and those for sexual hallucinations are 150 times higher than in the general population. The burden caused by hallucinations in general was substantial for most of the participants. The prevalence rate for childhood trauma was a full 100% in our sample. Our findings add to the existing body of published studies which challenge the traditional notion that hallucinations are rare and self-limiting in BPD. They thereby urge us to raise awareness of these phenomena, improve recognition, and develop evidence-based treatment methods, including those for trauma.

## Data Availability

The original contributions presented in the study are included in the article/**Supplementary Material**. Further inquiries can be directed to the corresponding author.
