# Supplementary material for: Sexual hallucinations and delusions in borderline personality disorder
Source: Front Psychiatry. 2026 Feb 25;17:1750041. doi: 10.3389/fpsyt.2026.1750041 (PMC12975991; doi:10.3389/fpsyt.2026.1750041)
Supplement: Supplementary file 1 [file DataSheet1.pdf]

Sexual Hallucinations and Delusions Questionnaire (SHDQ)

Translation of the Dutch *Vragenlijst Seksuele Hallucinaties en Wanen* (2018) by J.D. Blom, 2025

Patient number:
Date:
Researcher:

**Symptom**	**No**	**Yes, present state (last month)**	**Yes, lifetime**
1. Delusion of being watched in the shower			
2. Delusion of being watched in the bathroom			
3. Delusion of being watched while undressing			
4. Delusion of being watched in a different setting, please describe…			
5. Sexual delusions in which themes such as jealousy or infidelity of a loved one play a role			
6. Delusion of a sexual metamorphosis,<br>a. Of no longer being a man or a woman<br>b. Of being a neuter or eunuch<br>c. Of having swapped gender: man to woman or woman to			

man			
7. Delusion regarding changes to the size or shape of genitals			
8. Delusion of being followed or persecuted for sexual reasons			
9. Delusion regarding the sexual behaviour of others			
10. Delusion regarding one's own sexual behaviour			
11. Delusion about body parts and their movements taking on a sexual connotation			
12. Delusion of being pregnant			
13. Tactile hallucination (sensation of being touched inappropriately)			
14. Tactile hallucination (being abused, being sexually harrassed)			
15. Tactile hallucination			

(sensation of fluids on the skin, with a sexual connotation)			
16. Visual hallucination (seeing the face of an ‘abuser’)			
17. Visual hallucination (seeing the body image of an ‘abuser’)			
18. General visual hallucination with a sexual content			
19. Auditory hallucination with a sexual content			
20. Auditory hallucination of the voice of an ‘abuser’ or other characteristics (such as footsteps)			
21. Olfactory hallucination with sexual connotations			
22. Gustatory hallucination with sexual connotations			
23. Genital hallucinations			
24. Erotic sensations			

in other body parts			
25. Inappropriate sexual arousal			
26. Inappropriate orgasmic sensations			
27. Other sexually laden psychotic symptoms; if present, please describe…			
